# Postnatal corticosteroid exposure in very preterm infants: A French cohort study

**DOI:** 10.3389/fphar.2023.1170842

**Published:** 2023-04-05

**Authors:** Silvia Iacobelli, Käliani Allamèle-Moutama, Simon Lorrain, Béatrice Gouyon, Jean-Bernard Gouyon, Francesco Bonsante, the Logipren Collaborative Working Group

**Affiliations:** ^1^ Néonatologie, Réanimation Néonatale et Pédiatrique, CHU Saint Pierre, Saint Pierre, France; ^2^ Centre d’Etudes Périnatales de l’Océan Indien (UR 7388), Université de la Réunion, Saint Pierre, France

**Keywords:** drugs, electronic prescribing decision support system, database systems, chronic lung disease, benchmarking, neonates perinatal exposure, 1000 days

## Abstract

**Background:** Postnatal corticosteroids (PC) are widely used in very preterm infants. International reports and national multicenter trials describe a marked variability across countries and inter-sites, in the use of PC. Few information is available on therapeutic indications and prescription characteristics of PC.

**Aim:** The main objective of this study was to describe the exposure to PC in a large cohort of preterm infants born at less than 32 weeks of gestation, according to the prescription data of 41 tertiary-care NICUs in France. Secondary objectives were to describe therapeutic indications, day of life (DOL) of the first exposure, route of administration, duration, cumulative dose for each drug, and differences in exposure rates across centers.

**Methods:** We conducted a prospective observational cohort analysis from January 2017 to December 2021, in 41 French tertiary-care NICUs using the same computerized order-entry system.

**Results:** In total, 13,913 infants [birth weight 1144.8 (±365.6) g] were included. Among them, 3633 (26.1%) were exposed to PC, 21.8% by systemic and 10.1% by inhaled route. Within the study population, 1,992 infants (14.3%) received the first corticosteroid treatment in the first week of life and 1641 (11.8%) after DOL 7. The more frequent indications were prevention and/or treatment of bronchopulmonary dysplasia, and arterial hypotension. Hydrocortisone was the more often prescribed molecule. For systemic PC the first exposure occurred in mean at DOL 9.4 (±13.5), mean duration of treatment was 10.3 (±14.3) days, and the cumulative dose (expressed as the equivalent dose of hydrocortisone) was in median [IQR] 9.0 [5.5–28.8] mg/kg. For inhaled PC, the first exposure occurred in mean at DOL 34.1 (±19.7), and mean duration of treatment 28.5 (±24.4) days. The exposure rate ranged from a minimum of 5% to a maximum of 56% among centers, and significantly increased over the study period (*p* < 0.0001).

**Conclusion:** In this French cohort of very preterm infants, around one patient out to five was exposed to PC during hospital stay in the NICU. The exposure occurred early, starting from the first week of life. Exposure rate widely varied among centers. Pharmacoepidemiology studies are useful to increase knowledge on corticosteroid utilization patterns in preterm infants.

## Introduction

Corticosteroid drugs are medications with several therapeutic properties, including anti-inflammatory, anti-allergic, immunosuppressive and cortisol-mimicking effects. Postnatal corticosteroids (PC) represent one of the most common classes of drugs administered to sick neonates, the more so in very low birth weight (VLBW) infants. According to a recent cohort study conducted in 30 Neonatal Intensive Care Units (NICUs) in the US, the rate of exposure to at least one corticosteroid (among dexamethasone, prednisolone and methylprednisolone) reached 38% in extremely low birth weight (ELBW) infants (Puia-Dumitrescu et al., 2022). A pharmacoepidemiology report covering 29 French NICUs showed that hydrocortisone was one of the top five off-label (unapproved indication or posology) medications prescribed in preterm infants born at less than 28 weeks of gestation (Gouyon et al., 2019). In these children, PC are widely used due to their anti-inflammatory effects, for the prevention and/or the treatment of bronchopulmonary dysplasia (BPD) (Doyle, 2021), for earlier weaning of mechanical ventilation, as well to facilitate extubation (Khemani et al., 2009). Corticosteroid drugs are also prescribed in the treatment of refractory hypotension in preterm infants, as they increase cortisol concentration and responsiveness to circulating cathecolamines (Ibrahim et al., 2011).

Some PC may have adverse effects, including on the developing brain. The benefit/risk ratio of PC administration may change according to the chosen molecule, the route of administration, and the infant day of life (DOL), gestational age (GA) and morbidity risk. Based on the best knowledge available, some molecules should be avoided, exposure should be minimized, and systemic corticosteroids during the first week of life should be only used in very preterm infants at high risk of BPD (Jarreau et al., 2010; Doyle, 2021; Doyle et al., 2021). However, several reports describe a marked variability across countries, and inter-sites, in the use of PC, even after adjustment for infant characteristics (Nuytten et al., 2017; Puia-Dumitrescu et al., 2022). In these studies, the indications for use of corticosteroids in each infant are not available.

The main objective of this study was to describe the exposure to PC in a large cohort of preterm infants born at less than 32 weeks of gestation, according to the prescription data of 41 tertiary-care NICUs in France. Secondary objectives were to describe: 1) the therapeutic indications; 2) the time-point of the first exposure; 3) the route of administration, duration and cumulative dose for each drug; 4) the eventual differences in exposure rates across centers.

## Materials and methods

### Study design and setting

This was an exploratory observational study carried out in 41 tertiary-care NICUs in France, from January 2017 to December 2021.

### Study population and inclusion criteria

All infants born at less than 32 weeks of gestation, and admitted on their first DOL in one the 41 NICUs using the same computerized order-entry system (CPOE), Logipren^®^, were eligible. As indicated by a recent report, there are 68 NICUs in France (Data.drees.solidarites-sante.gouv.fr, 2023), thus this study examines 60% of French NICUs.

### Data sources

The CPOE associated with a Clinical Decision Support System (Logipren^®^) has been described in previous studies (Gouyon et al., 2017; Gouyon et al., 2019). This CPOE allows medication prescription according to GA, postnatal age, post-conceptional age, body weight at the day of the prescription, and indication. The system provides a complete preselected drug prescription with drug dose, modality of administration, preparation modalities and warnings. The prescriber has to indicate the daily body weight, choose the drug, specify its indication, and modify or confirm the prescription after a warning. The database also provides information on drug adverse effects, which are recorded on the last day of treatment. At the time of prescription, all electronic prescriptions are prospectively and automatically stored on local computer servers. After full anonymization (deidentification) within each participating hospital, they are sent to the same data warehouse for subsequent analysis.

### Outcomes

The main outcome of interest was PC exposure. Exposure was defined as having at least one dose of any corticoid drug administered at any time during the hospital stay. No exposure was defined if there were no documented doses administered during that time. The PC of interest (systemic or inhaled administration) were hydrocortisone, betamethasone, prednisolone, methylprednisolone, prednisone, dexamethasone, budesonide, fluticasone.

Secondary outcomes were the following: therapeutic indication; time of the first exposure, expressed as DOL of the first corticosteroid administration; duration of exposure, classified by total days of exposure; cumulative dose (mg/kg) of hydrocortisone and methylprednisolone. For systemic PC, the total cumulative corticosteroid dose was calculated as the sum of all doses of any intravenous or oral corticosteroid, expressed as the equivalent dose of hydrocortisone (mg/kg). The frequency and the type of PC side effects were analysed. Variations in exposure rates across centers were assessed.

### Statistical analysis

Categorical variables were presented as numbers and proportions. Continuous variables were presented as mean ± standard deviations or median and range. Normality was checked using the Shapiro-Wilk test. The 95% confidence intervals (95% CI) were calculated for the main outcome. Cochran–Armitage test for trend was applied. Bivariate comparisons were performed using χ2 test, or Student test for qualitative variables. A *p*-value below 0.05 was considered significant. Statistical analysis was conducted using SAS^®^ software (Version 9.4, SAS Institute, North Carolina, United States).

## Results

### Population characteristics and PC exposure rate

During the study period, the CPOE database recorded 16,363 neonates born before 32 weeks of gestation. Among them, 2,450 were not eligible, as they were admitted in the NICU after DOL 1. In final, 13,913 newborn infants were retained for the analysis and represented the study population. Among them, 3,633 (26.1%) were exposed to at least one corticosteroid (Table 1). Overall, the exposure rate varied according to GA, the lower the GA, the higher the exposure rate.

**TABLE 1:** Characteristics of the study population and postnatal corticosteroid exposure rate.

		Gestational age (weeks of gestation)		
	Population *n* = 13913	(24–25) *n* = 1780	(26–27) *n* = 2954	(28–31) *n* = 9179
Male, n (%)	7407 (53.2)	985 (55.3)	1511 (51.2)	4911 (53.5)
Birth weight (g), mean (±SD)	1144.8 (365.6)	708.5 (117.2)	874.7 (188.6)	1316.5 (314.1)
Length of stay (days), mean (±SD)	42.4 (32.0)	51.6 (45.8)	55.8 (37.0)	36.3 (24.4)
Mortality, n (%)	1415 (10.2)	649 (36.5)	437 (14.8)	329 (3.6)
PC exposure, n (%) [CI 95%]	3633 (26.1) [25.4; 26.8]	1232 (69.2) [67.1; 1.4]	1500 (50.8) [49.0; 2.6]	901 (9.8) [9.2; 10.4]

PC, postnatal corticosteroids; CI, confidence interval.

Within the study population, 1,992 infants (14.3%) received the first corticosteroid treatment in the first week of life and 1,641 (11.8%) after DOL 7. The most frequent indication for PC during the first week of life was BPD prevention. The treatment of BPD was the main indication for PC after DOL 7. Hydrocortisone was the most prescribed corticosteroid drug throughout the hospital stay. These data are shown on Table 2.

**TABLE 2:** Time-point exposure, route of administration, indications and corticosteroid drugs used in the study population.

		Gestational age (weeks of gestation)		
	Population *n* = 13913	(24–25) *n* = 1780	(26–27) *n* = 2954	(28–31) *n* = 9179
DOL at the first PC exposure				
Not exposed	10280 (73.9)	548 (30.8)	1454 (49.2)	8278 (90.2)
DOL 1–7	1992 (14.3)	771 (43.3)	837 (28.3)	384 (4.2)
DOL 8–14	333 (2.4)	133 (7.5)	140 (4.7)	60 (0.7)
DOL 15–21	404 (2.9)	124 (7.0)	172 (5.8)	108 (1.2)
DOL 22–28	470 (3.4)	129 (7.2)	186 (6.3)	155 (1.7)
DOL >28	434 (3.1)	75 (4.2)	165 (5.6)	194 (2.1)
Route of administration, n (%)				
Systemic	3029 (21.8)	1172 (65.8)	1261 (42.7)	596 (6.5)
Intravenous	2821 (20.3)	1121 (63.0)	1171 (39.6)	529 (5.8)
Oral	682 (4.9)	251 (14.1)	310 (10.5)	121 (1.3)
Inhaled	1411 (10.1)	414 (23.3)	567 (19.2)	430 (4.7)
Therapeutic Indications (DOL 1–7), n (%)				
BPD prevention	1081 (7.7)	452 (25.4)	539 (18.2)	90 (0.9)
Hypotension	845 (6.1)	308 (17.3)	284 (9.6)	253 (2.8)
BPD treatment	35 (0.3)	7 (0.4)	15 (0.5)	13 (0.1)
Facilitate extubation	6 (0.0)	4 (0.2)	0 (0.0)	2 (0.0)
Obstructive dyspnoea	6 (0.0)	1 (0.1)	3 (0.1)	2 (0.0)
Other	175 (1.3)	58 (3.3)	65 (2.2)	52 (0.6)
INN (DOL 1–7), n (%)				
Hydrocortisone	1950 (14.0)	763 (42.9)	822 (27.8)	362 (4.0)
Budesonide	42 (0.3)	8 (0.4)	17 (0.6)	17 (0.2)
Betamethasone	12 (0.1)	3 (0.2)	6 (0.2)	3 (0.0)
(Methyl-)Prednisolone	2 (0.0)	1 (0.1)	0 (0.0)	1 (0.0)
Therapeutic Indications (DOL > 7), n (%)				
BPD treatment	1753 (12.6)	637 (35.8)	725 (24.5)	391 (4.3)
BPD prevention	957 (8.4)	418 (23.5)	442 (20.5)	75 (0.8)
Hypotension	626 (4.5)	273 (15.3)	236 (8.0)	117 (1.3)
Facilitate extubation	84 (0.6)	38 (2.1)	37 (1.3)	9 (0.1)
Obstructive dyspnoea	62 (0.4)	20 (1.1)	18 (0.6)	24 (0.3)
Other	969 (7.0)	335 (18.8)	370 (12.5)	264 (2.9)
INN (DOL > 7), n (%)				
Hydrocortisone	1637 (11.8)	665 (37.4)	740 (25.1)	232 (2.5)
Budesonide	1298 (9.3)	400 (22.5)	524 (17.7)	374 (4.1)
Betamethasone	887 (6.4)	389 (21.9)	368 (12.5)	130 (1.4)
Fluticasone	298 (2.1)	86 (4.8)	121 (4.1)	91 (1.0)
Methylprednisolone	45 (0.3)	12 (0.7)	24 (0.8)	9 (0.1)
Dexamethasone	6 (0.0)	1 (0.1)	2 (0.1)	3 (0.0)

DOL, day of life; PC, postnatal corticosteroids; INN, international non-proprietary name of drugs.

The exposure rate of the study population varied from 23.6% to 28.0%, and there was a significant trend toward increased exposure rate over time (*p* < 0.0001).

There were key differences between exposed and not-exposed infants, regarding sex, GA, birth weight, length of hospitalization and mortality (data shown in Table 3).

**TABLE 3:** Comparison of exposed and non-exposed infants.

	Study population		
	Exposed n = 3633	Not-exposed *n* = 10280	*p*-value
Male, n (%)	2077 (57.2)	5330 (51.8)	<0.0001
Birth weight (g), mean (±SD)	871.0 (270.5)	1241.6 (345.3)	<0.0001
Gestational age (weeks), mean (± SD)	26.5 (1.8)	29.0 (1.8)	<0.0001
Length of stay (days), mean (± SD)	59.9 (40.5)	36.2 (25.7)	<0.0001
Mortality, n (%)	794 (21.9)	621 (6.0)	<0.0001

#### Other exposure variables of interest

Adverse effects were recorded for 46 (1.3%) of the exposed patients. The most frequent side effect was arterial hypertension (*n* = 17), followed by spontaneous gastrointestinal perforation (*n* = 8), hypertrophic cardiomyopathy (*n* = 5) and hyperglycaemia (*n* = 3).

Table 4 shows the postnatal age at the first systemic PC exposure, the duration of treatment for systemic PC and the total cumulative corticosteroid dose.

**TABLE 4:** Characteristics of systemic postnatal corticosteroid administration in exposed infants.

		Gestational age (weeks of gestation)		
	Exposed to systemic PNC *n* = 3029	(24–25) *n* = 1172	(26–27) *n* = 1261	(28–31) *n* = 596
First exposure, DOL				
Mean (±SD)	9.4 (13.5)	8.4 (11.6)	9.7 (13.3)	12.2 (16.8)
Median [p25%–p75%]	2 [1–15]	2 [1–14]	2 [1–14]	4 [2–19]
Min–max	1–127	1–110	1–127	1–103
Duration of treatment for systemic PC, days				
Mean (±SD)	10.3 (14.3)	12.1 (16.1)	10.2 (13.0)	7.3 (12.2)
Median [p25%–p75%]	7 [3–11]	7 [3–14.5]	9 [3–11]	3.5 [2–8]
Min–max	1–190	1–176	1–190	1–132
Cumulative dose of systemic PC^a^, mg/kg				
Mean (±SD)	23.4 (35.2)	27.2 (35.2)	22.0 (33.9)	19.1 (37.4)
Median [p25%–p75%]	9.0 [5.5–28.8]	12.0 [6.0–36.7]	9.0 [6.0–25.7]	8.0 [3.5–16.8]
Min–max	0.0–396.1	0.0–281.8	0.0–362.7	0.0–396.1
Number of different INN, n (%)				
1	2575 (85.0)	949 (81.0)	1067 (84.6)	559 (93.8)
2	426 (14.1)	217 (18.5)	175 (13.9)	34 (5.7)
3	28 (0.9)	6 (0.5)	19 (1.5)	3 (0.5)

DOL, day of life; PC, postnatal corticosteroids; INN, international non-proprietary name of drugs.

^a^ Calculated as the sum of all doses of any intravenous or oral corticosteroid, expressed as the equivalent dose of hydrocortisone.

Table 5 details the time-point of the first administration, the duration of treatment and the total dose for the most prescribed systemic drugs, hydrocortisone and betamethasone.

**TABLE 5:** Characteristics of hydrocortisone and betamethasone administration in exposed infants.

	Hydrocortisone *n* = 2565	Betamethasone *n* = 893
First exposure, DOL		
Mean (±SD)	6.7 (11.8)	27.9 (17.4)
Median [p25%–p75%]	2 [1–7]	23 [19–30]
Min–max	1–120	1–165
Duration of treatment, days		
Mean (±SD)	9.1 (13.3)	8.9 (9.4)
Median [p25%–p75%]	6 [2–10]	6 [3–10]
Min–max	1–188	1–110
Cumulative dose, mg/kg		
Mean (±SD)	14.5 (23.7)	1.3 (1.4)
Median [p25%–p75%]	8 [4–12.5]	0.9 [0.5–1.8]
Min–max	0.1–260	0.1–12.7

DOL, day of life.

Within the study population, 1,411 infants were exposed to aerosolized PC (budesonide, fluticasone, and dexamethasone). More precisely, 1,327 infants were exposed to budesonide, 298 were exposed to fluticasone, and 5 to dexamethasone. Thus, the most prescribed drug was budesonide, administered to 94% of infants exposed to inhaled corticosteroids. The main indications for inhaled PC were BPD treatment, and adjuvant for extubation (respectively 79.5% and 44.1% of exposed infants). Other exposure variables of interest in infants receiving aerosolized PC are illustrated in Table 6.

**TABLE 6:** Exposure variables of interest for inhaled PC.

		Gestational age (weeks of gestation)		
	Exposed to inhaled PC *n* = 1411	(24–25) *n* = 414	(26–27) *n* = 567	(28–31) *n* = 9179
First exposure, DOL				
Mean (±SD)	34.1 (19.7)	36.9 (21.2)	34.1 (21.2)	31.2 (15.3)
Median [p25%–p75%]	29 [22–40]	31 [23–44]	29 [22–39]	28 [22–39]
Min–max	[1–184]	[1–119]	[1–184]	[1–97]
Duration of treatment, days				
Mean (±SD)	28.5 (24.4)	35.6 (28.8)	30.1 (24.0)	19.7 (16.2)
Median [p25%–p75%]	22 [11–39]	28.5 [14–52]	23 [14–43]	16 [8–28]
Min–max	[1–161]	[1–152]	[1–161]	[1–95]
Number of different INN, n (%)				
1	1192 (84.5)	335 (80.9)	480 (84.7)	376 (87.4)
2	219 (15.5)	79 (19.1)	87 (15.3)	54 (12.6)

#### Exposure rate across centers

**FIGURE 1:**
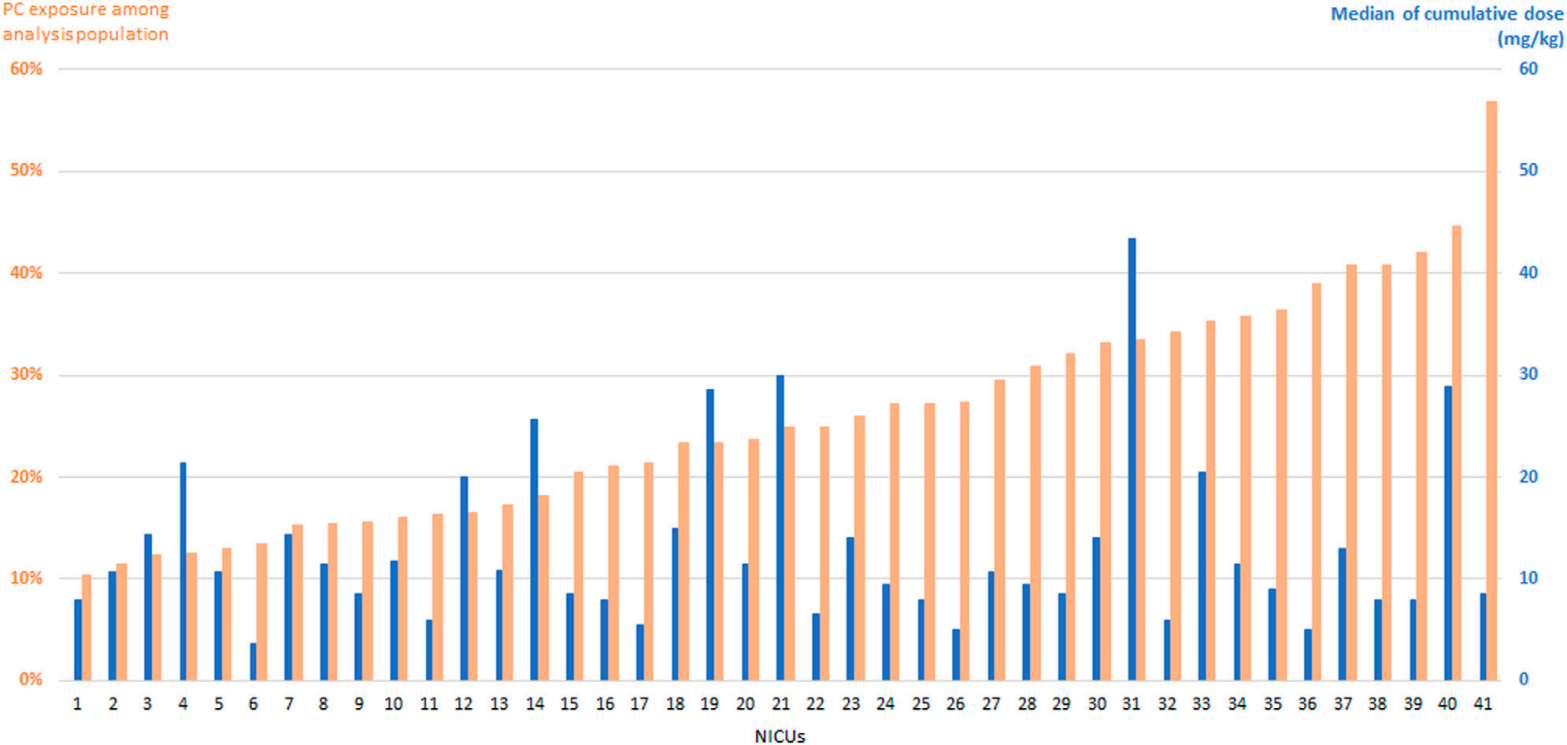
Postnatal corticosteroids (PC) exposure rate in infants born at less than 32 weeks of gestation (orange bars). The blue bars indicate the median cumulative dose (mg/kg) of systemic PC in each center.

The exposure rate to PNC varied from 5% to 56% across centers (Figure 1). In general, the exposure rate in each center was not correlated to the proportion of infants born between 24 and 27 weeks of gestation (data not shown). Variations in median cumulative dose of systemic PC among centers is illustrated in Figure 1.

## Discussion

This study describes the corticosteroid prescription practice over a 5-year period in a French cohort of very preterm infants. In this cohort, the exposure rate to PC was 26.1%, and more precisely 21.8% for systemic and 10.1% for inhaled administration. Exposed infants, compared with unexposed ones, had a lower birth weight, were born earlier, and were more often male, as in previous studies (Puia-Dumitrescu et al., 2022). This is likely because these variables are recognized risk factors associated with the occurrence of BPD (Ramos-Navarro et al., 2022). Actually, we found that the main indication for corticosteroid use in our cohort was BPD treatment.

To our knowledge, this is the first study investigating the real exposure rate to corticosteroids in a large longitudinal cohort of preterm infants cared for in NICUs, in France. The exposure rate found in our study population is higher than that reported in the multicenter cohort EPICE (Nuytten et al., 2017), where the overall exposure rate to PC was 13.9%. Three French tertiary-care NICUs had been included in that European study, and they displayed an exposure rate varying from 10% to 15%, in infants born at less than 29 weeks of gestation. In contrast to our study, the results of the EPICE cohort were based on questionnaires extracted from neonatal records, and on self-declared policies concerning PC prescription. This method may have underestimated actual administrations.

Our results showed a significant trend towards increasing the use of PC from 2017 to 2021 in French NICUs, and found that the first exposure to corticosteroids occurred more often during the first week of life. This can be explained by the results of the multicenter, national randomized controlled trial (RCT) published in 2017 by Baud et al. (2016). This study, published after the results of the EPICE cohort, had proven the efficacy of early hydrocortisone administration in preventing BPD in ELBW infants (high to moderate quality of evidence). Moreover, subsequent systematic reviews and follow-up reports have shown favourable results in terms of survival without BPD (Shaffer et al., 2019) and mean-term neurodevelopmental outcome, following early exposure to hydrocortisone (Peltoniemi et al., 2009; Baud et al., 2017). This molecule was the most used corticosteroid drug in our cohort (18.4%). However, noteworthy is the fact that in 12.7% of cases it was used with the indication “BPD treatment.” Nevertheless, two recent RCT showed no benefit of later, higher-dose of hydrocortisone, to treat established or evolving BPD (Onland et al., 2019; Watterberg et al., 2022).

National recommendations for the use of corticosteroids in infants with VLBW were last updated in 2010 in France (Jarreau et al., 2010) (ANSM, 2010). The French Agency of Drug Safety (ANSM, 2010) discourages early betamethasone administration, and suggests not administering this molecule before the third postnatal week of life. Systemic dexamethasone is not recommended for the prevention or treatment of chronic lung disease in preterm infants. The conduct of betamethasone and dexamethasone prescriptions seems consistent with these guidelines in our cohort. Indeed, betamethasone was used in 6.4% of the study population, and the mean time-point of the first administration was at 27.6 DOL. The first administration time of betamethasone in our study is consistent with recent findings on the association between timing of systemic PC and severity of BPD (Harmon et al., 2020; Cuna et al., 2021). Results from two retrospective cohorts of very (Cuna et al., 2021) and extremely (Harmon et al., 2020) preterm infants, showed that later treatment (after DOL 36 and DOL 50 respectively) was associated with increased likelihood to develop severe BPD, compared to earlier treatment.

Our results showed an extremely low exposure to dexamethasone, as only six infants within the study population received this drug. These data are different from those of the EPICE cohort, showing exposure rates of 35.8% (Shaffer et al., 2019), and from those reported in some US studies (up to 38% in extremely preterm infants) (Puia-Dumitrescu et al., 2022). In the retrospective study of the UK Neonatal Collaborative (Yao et al., 2022), the rate of PC use for the prevention or treatment of BPD was 8% in 62,019 infants born at less than 32 weeks of gestation, thus substantially lower compared to our cohort. However, despite an overlapping between the study periods, it is difficult to compare these populations, as in the UK cohort dexamethasone was predominantly used, followed by late hydrocortisone.

In our study, the treatment durations for hydrocortisone and betamethasone were lower, compared to other works (Nuytten et al., 2017). Again, it is not possible to compare our data with previous studies with regard to cumulative doses, since we expressed the cumulative dose of systemic PC as the equivalent dose of hydrocortisone, and this information is not available from previous reports (Puia-Dumitrescu et al., 2022; Douglas et al., 2023).

The rate of adverse effects in our cohort was 1.3%. This rate is low, but it is likely that CPOE LOGIPREN^®^ underestimate side effects, as these can be reported only on the day when the drug prescription is withdrawn. Non-etheless, adverse effects may occur several days after stopping the treatment (Doyle, 2021).

In our cohort, around 10% of infants were exposed to inhaled corticosteroids. The first exposure occurred on average after the first week of life. This practice is not consistent with the finding that no significant benefits have emerged to date, based on available data on treatment with inhaled PC starting after DOL 7 (Onland et al., 2017; Filippone et al., 2019). The mean total duration of budesonide use was 28.5 days, thus slightly lower than in the trial of Bassler et al. (2015), studying the short-term and long-term efficacy and safety of early inhaled budesonide for the prevention of BPD in extremely preterm infants. This RCT had found higher mortality rate among neonates who received budesonide compared to placebo. This information was not available in the present study, as we did not analyse prespecified secondary outcomes associated with inhaled corticosteroids.

As expected, there was a great variability in corticosteroid exposure and in median cumulative dose of systemic PC across sites. Interestingly, we did not find a link between exposure rates and patient severity (as estimated based on the proportion of extremely preterm infants cared for in each center). This leads us to the hypothesis that administration practice of corticosteroids is still based on individual choices, and not necessarily on the patient needs. However, this interpretation cannot be confirmed by our results, as risk factor variables and clinical scores of illness were not available in this cohort. In general, we acknowledge that the present study would have benefit from the analysis of a greater number of clinical covariates and outcomes (in particular, the occurrence of BPD), which were not available in the cohort. This is one limitation of our research. Another limitation is the lack of information on prenatal corticosteroid administration, which is also a factor that positively influences the prognosis of premature infants, as it reduces the severity and the occurrence of respiratory distress, and therefore the need for PC. Our study has several strengths. Due to the high number of included sites, our cohort can be considered a nationwide representative sample of practice in NICUs. In addition, the strength of this study also lays on the large number of the included infants, and on the exhaustive information available, regarding prescriptions. Since all included units participate to a benchmarking program of their prescriptions, data collection is prospective, rigorous, homogeneous and corresponding to real prescriptions. We finally note that the number of missing data is negligible in our database.

## Conclusion

This study described the corticosteroid exposure rate and prescription practice in a French cohort of very preterm babies. The results allow us to conclude that the use of corticosteroids remains frequent in NICUs, as one out of every five very preterm infants is exposed to at least one corticosteroid drug during the hospital stay. This exposure occurs early, starting from the first week of life. Adherence to national guidelines and practice implementation regarding warnings on PC use are satisfying. The increasing use of hydrocortisone in the prevention of BPD reflects results of recent studies on this molecule. These results argue for continuation of long-term follow-up and optimization of corticosteroid use in vulnerable infants.

## Logipren collaborative working group

S. Abasse, CH Mayotte; C. Alexandre, CHU Caen; R. Brat, CHR Orléans; D. Brault, CH Argenteuil; L. Brisseau, CH Vannes; P. Boize, CH Pontoise; Y. Couringa, CH Cayenne; F. Decobert, CHI Créteil; C. Desbruyeres, CH Chambéry; M. Dorsi, CHT Nouméa; A. Elgellab, CH Lens; G. Escourrou, CHI Montreuil, F. Flamein, CHU Lille; O. Flechelles, CHU Martinique; G. Ghostine, CHU Amiens; O. Girard, CH St-Denis; E. Jobard, CH Saint-Brieuc; L. Karaoui, CH Meaux; E. Kermorvant-Duchemin, Hôpital Necker AP-HP; A. Kieffer, CH Le Mans; F. Kieffer, CH Trousseau AP-HP; C. Lafon, CH Arras; S. Lebouedec, CHU Angers; D. Leger, CH Basse-Terre; I. Ligi, AP-HM Conception; M. Di Maio, CHU Nîmes; G. Mazeiras, CH Cote-Basque; F. Michel, AP-HM Timone; D. Mitanchez, CHU Tours; F. Mons, CHU Limoges; J. Mourdie, CH Le Havre; A. Moussy-Durandy, CHI Poissy; C. Nicaise, AP-HM Hôpital Nord; K. Norbert, CH Pau; A. S. Pages, CH Cotentin; D. Ramful, CHU Nord-Réunion; H. Razafimahefa, CH Corbeil-Essonnes; J. M. Rosenthal, CHU Guadeloupe; C. Tripon, CHU Poitiers; M. Vidal, CH Perpignan.

## Data Availability

The raw data supporting the conclusion of this article will be made available by the authors, without undue reservation.
